# Natural Death

**Published:** 1885-11

**Authors:** 


### Natural Death,

Is to die sweetly without a sob, a struggle or a sigh. It is the result of a long life of uninterrupted health, of a long life of "*temperance in all things,*" and such a death should be one of the ends and aims of every human being, so that we may not only live long, but in that long life be able to do much for man, and much for God.

The love of life is a universal instinct; life is a duty, its peril or neglect a crime. We are placed on earth for a purpose, that purpose can be none other than to give us an opportunity of doing good to ourselves and to others, and to be anxious to be "*off duty*," sooner than God wills, is no indication of true piety. The good man has one ruling, ever present desire, and that is to live as long on the earth as his maker pleases, and while living to do the utmost he can to benefit and bless mankind ; and to accomplish a long and active, and useful life, and study how to preserve and promote a high degree of bodily health is indispensable. And it seems to have been ordained by a Providence both kind and wise, as a reward of a temperate life, that such a life should be largely extended, and that its decline should be as calm as a summer's evening, as gentle as the babe sleeps itself away on its mother's bosom.

These sentiments were advocated in one of the first numbers of the Journal in the way of argument to induce its readers to live temperately in all things, in order by length of life to secure the largest leisure for doing the greatest amount of good.

